# Complete mitochondrial genome of *Cyclograpsus intermedius* Ortmann, 1894 (Crustacea: Decapoda: Grapsoidea) specimen collected in South Korea

**DOI:** 10.1080/23802359.2020.1842265

**Published:** 2021-01-05

**Authors:** Jiyeong Shin, Sang Chul Choi, Jongwoo Jung

**Affiliations:** a The Division of EcoCreative, Ewha Womans University, Seoul, Korea; b Department of Biotechnology, Sungshin Women’s University, Seoul, Korea; c Department of Science Education, Ewha Womans University, Seoul, Korea

**Keywords:** *Cyclograpsus intermedius*, phylogenetic analysis, crab, mitochondrial genome

## Abstract

In this study, the complete 16,184 bp mitochondrial genome of *Cyclograpsus intermedius* was determined from a specimen collected in South Korea. It consists of 13 protein-coding, 22 tRNA, 2 rRNA genes, and a non-coding A + T rich region. The base composition of the heavy strand in the mitochondrial genome was 34.7% A, 10.7% G, 18.7% C, and 35.9% T, resulting in a G + C content of 29.4%. A maximum-likelihood phylogenetic tree based on the 13 mitochondrial protein-coding genes showed that *C. intermedius* clustered together with the Varunidae. These molecular data will be useful for studying the evolutionary relationships among crab species.

Grapsoidea is a superfamily of crabs comprising species that adapt to terrestrial, semi-terrestrial, or freshwater environments and plays an important role in the coastal ecosystem (Lee 1998). The species *Cyclograpsus intermedius* inhabits both temperate and tropical regions, including Korea, Japan, Taiwan, and the Indian Ocean (Hangai et al. 2009; Tan et al. 2016). Despite the discovery of morphologically similar species in the genus *Cyclograpsus*, genetic and taxonomic features of the genus remain uncharacterized (Griffin 1968; Hangai et al. 2009). We sequenced the mitochondrial genome of a Korean specimen of *C. intermedius* to construct the taxonomy and phylogeny of grapsid crabs; thus, providing more molecular data relating to this superfamily of crabs (GenBank accession number: MT621398).

A specimen of *C. intermedius* was collected from the rocky intertidal zone of Dokdo, South Korea on 20 September 2019 (geographic location: 37°14′29.6′′ N, 131°52′10.3′′ E). The specimen was preserved in 80% ethanol and stored at the Ewha Womans University Natural History Museum in Korea (accession number: EWNHMMAR767). Total DNA was extracted from the muscle of the dissected walking legs of the specimen using DNeasy Blood & Tissue (Qiagen, Valencia, CA). The mtDNA was sequenced using the Novaseq 6000 System (Illumina, San Diego, CA). The MITObim method (Hahn et al. 2013) and MITOS (Bernt et al. 2013) were used for the assembly and annotation of the complete mitochondrial genome, respectively.

The mitogenome of *C. intermedius* was 16,184 bp in length, which is a typical length of Decapoda mitogenomes. It included 13 protein-coding, 22 tRNA, 2 rRNA genes, and a non-coding A + T-rich control region. For the 13 protein-coding genes, the most common shared start codon was ATG (in *COX1*, *COX2*, *COX3, ATP8*, *ND4L,* and *ND4*), followed by ATT (*ATP6*, *ND5*, and *ND6*). The most common termination codon was TAA (*COX1, COX3, ND1, ND3*, *ND4*, *ND4L*, *ND6*, *ATP6*, and *ATP8*), followed by the incomplete termination codon T– (*ND2*, *ND5*, *CYTB*, and *COX2*). The overall mitochondrial base composition of this genome was A: 34.7%, T: 35.9%, G: 10.7%, and C: 18.7%, with a G + C content of 29.4%.

To determine the phylogenetic relationship of *C. intermedius,* a multiple sequence alignment was prepared by concatenating sequences of the 13 mitochondrial protein-coding genes from 11 crab species and an outgroup (*Harpiosquilla harpax*) in the NCBI GenBank database. A maximum-likelihood phylogenetic tree was constructed using MEGA X (Kumar et al. 2018) with 1000 bootstrap replicates (Figure 1). *Cyclograpsus intermedius* was clustered with other Varunidae species, suggesting that *C. intermedius* is also a member of Grapsoidea. In conclusion, the complete mitogenome of *C. intermedius* provides fundamental phylogenetic information of the genus *Cyclograpsus*.

**Figure 1.**
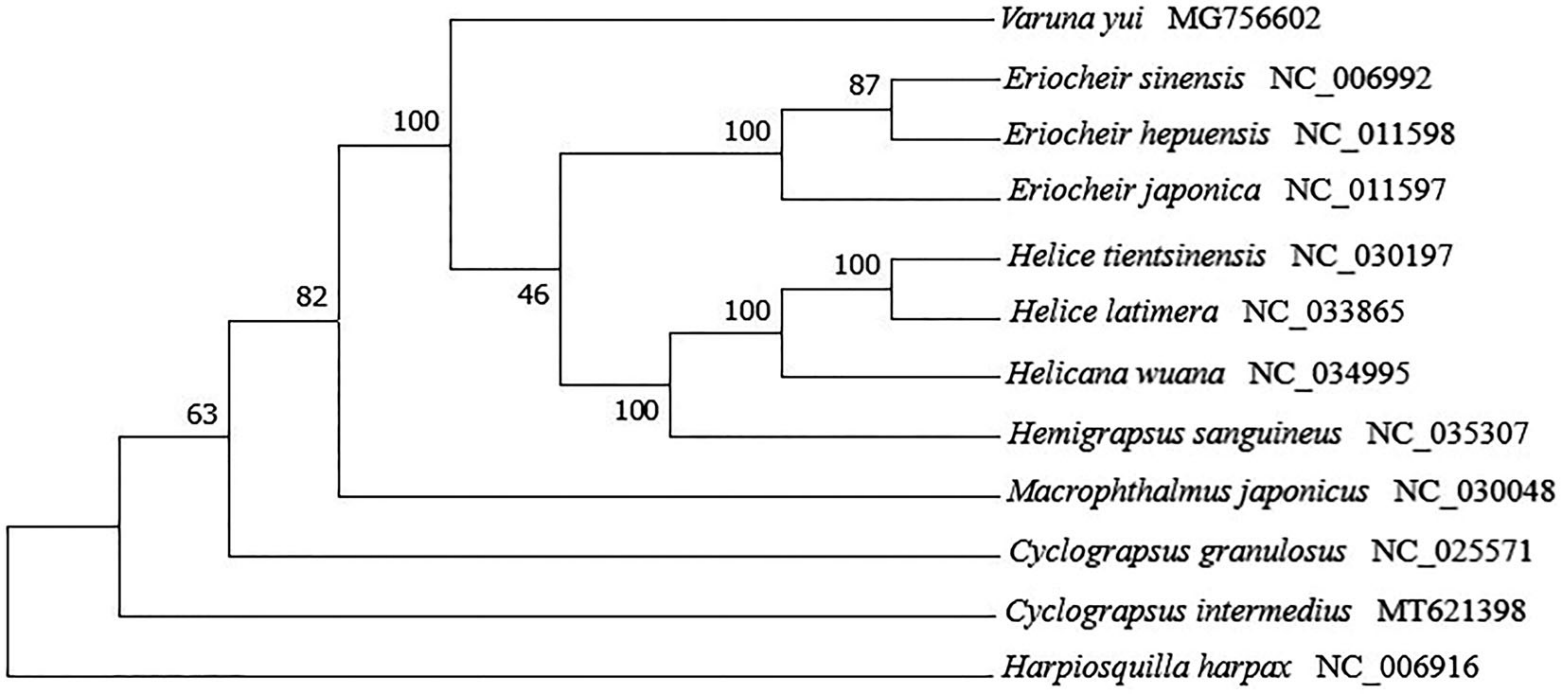
Phylogenetic tree of 11 crab species and an outgroup constructed using the maximum likelihood (ML) method based on 13 protein-coding genes.

## Data Availability

The data that support the findings of this study are openly available in Mendeley Data at http://dx.doi.org/10.17632/jn3wh7bc74.1
